# Quantifying societal burden of radiation-induced small bowel toxicity in patients with rectal cancer

**DOI:** 10.3389/fonc.2024.1340081

**Published:** 2024-07-08

**Authors:** Eva Kimpe, Riet Parmentier, Sara-Lise Busschaert, Johan De Mey, Kurt Barbé, Mark De Ridder, Koen Putman

**Affiliations:** ^1^ Interuniversity Centre for Health Economics Research (I-CHER), Department of Public Health, Vrije Universiteit Brussel, Brussels, Belgium; ^2^ Department of Radiology, Universitair Ziekenhuis Brussel, Vrije Universiteit Brussel, Brussels, Belgium; ^3^ Biostatistics and Medical Informatics Research Group (BISI), Department of Public Health, Vrije Universiteit Brussel, Brussels, Belgium; ^4^ Department of Radiotherapy, Universitair Ziekenhuis Brussel, Vrije Universiteit Brussel, Brussels, Belgium

**Keywords:** radiotherapy, rectal cancer, cancer survivors, cost-benefit analysis, health care economics and organizations

## Abstract

**Introduction:**

Advancements in rectal cancer (RC) treatment not only led to an increase in lives saved but also improved quality of life (QoL). Notwithstanding these benefits, RC treatment comes at the price of gastrointestinal morbidity in many patients. Health economic modelling poses an opportunity to explore the societal burden of such side-effects. This study aims to quantify radiation-induced late small bowel (SB) toxicity in survivors of RC for Three-Dimensional Conformal Radiation Therapy (3D-CRT), Intensity Modulated Radiation Therapy (IMRT) and Intensity Modulated Radiation Therapy – Image Guided Radiation Therapy (IMRT/IGRT).

**Materials and methods:**

Materials and A model-based health economic evaluation was performed. The theoretical cohort consists of a case-mix of survivors of RC aged 25-99 years according to Belgian age-specific incidence rates. A societal perspective was adopted. The base case analysis was complemented with one-way deterministic analyses, deterministic scenario analyses and probabilistic sensitivity analysis (1,000 iterations). Results were presented as mean lifetime incremental cost (€) and utility (QALYs) per patient.

**Results:**

The analyses showed that the use of innovative radiotherapy (RT) improves lifetime QoL in survivors of RC by 0.11 QALYs and 0.05 QALYs by preferring IMRT/IGRT and IMRT over 3D-CRT, respectively. The use of IMRT/IGRT and IMRT results in an incremental cost-saving of €3,820 and €1,863 per patient, solely by radiation-induced SB toxicity, compared to 3D-CRT.

**Discussion and conclusion:**

It is important to consider late toxicity effects in decisions regarding investments and reimbursement as our analysis highlighted the potential long-term cost-savings and improved QoL of novel RT techniques in patients with rectal cancer.

## 1 Introduction

Cancer survivorship is defined as “a process that begins at the moment of diagnosis and continues through the balance of life” (1). Miller et al. (2) refer to permanent survivors as cancer-free patients who are enduring from cancer and its treatment (2, 3). Post-treatment surveillance consists of several components including preventing and intervening with conditions resulting from cancer treatment itself (4).

A specific concern in radiotherapy (RT) of patients with rectal cancer (RC) is gastrointestinal morbidity. Late small bowel (SB) toxicity may occur months to years after completion of RT and induces long-lasting and even irreversible changes to the epithelium (5). Typically, radiation-induced SB toxicity manifests as dysmobility, stricture formation, malabsorption and diarrhea. In addition, bacterial overgrowth might be accompanied by bloating, excessive gas and borborygmi (5). In severe cases, patients might experience obstruction, bleeding or perforation which requires hospitalization for further observation or more invasive interventions (6, 7). Studies indicate that up to 20-30% of patients suffer from late SB toxicity (6, 8). Research demonstrated that these persistent symptoms impede on patient’s social functioning and therefore, affecting patients’ long-term quality of life (QoL) (8–11). Apart from personal burden, postoperative morbidities put pressure on healthcare resources as complications result in hospital readmissions associated with substantial intra- and extramural healthcare costs (12). The indirect economic cost of cancer mortality is well documented with an estimate of 0.58% (range: 0.25%-1.05%) of the European gross domestic product (13). Although the evidence suggests that gastrointestinal disorders are the main reason for increased healthcare utilization in survivors of RC, the economic impact of survivorship comorbidities seems less considered in such cost-of-illness studies (14).

Model-based health economic (HE) evaluations gain interest as outcomes are estimated over large time horizons whereas economic evaluations alongside randomized controlled trials are bounded to the follow-up period of the trial. Therefore, decision-analytic HE modelling offers a valuable solution to estimate long-term societal burden by comparing costs and quality-adjusted life years (QALYs) resulting from two or more strategies (15). Currently, these techniques are mainly applied in oncology to guide policy decisions concerning the implementation of cost-effective screening programmes or treatment strategies (16, 17). Furthermore, the little existing evidence evaluating long-term cancer survivorship care focuses on cancer recurrence rather than on treatment-induced morbidity and mortality such as SB toxicity (18).

In brief, the long-term societal burden resulting from mortality and morbidity which go beyond cancer treatment is less explored in the current literature. It is worthwhile to investigate the economic burden of SB toxicity in patients with RC given the increasing number of survivors and the prevalence of the symptoms in these patients (6, 8, 19). Therefore, this study aims on quantifying the societal cost of late SB toxicity resulting from past and current RT techniques in survivors of RC by applying conventional HE modelling methods.

## 2 Materials and methods

### 2.1 Study population, setting and study perspective

The cohort represents a closed cohort of resectable locally advanced RC survivors (cT3/4 or node-positive) whose treatment usually entails radiotherapy (10, 20). The simulated cohort includes patients aged 25-99 years according to their representation within Belgian age-specific incidence rates in 2019 (21). A limited societal perspective was adopted in which healthcare costs and indirect costs outside the healthcare sector, more specifically productivity loss and premature death due to SB toxicity, are included (22). The study protocol was approved by the ethical committee at the Universitair Ziekenhuis Brussel, Brussels, Belgium (B.U.N. 1432020000259). All analyses were performed using R Statistical Software (v4.1.2; R Core Team 2021).

### 2.2 Model structure

**Figure 1:**
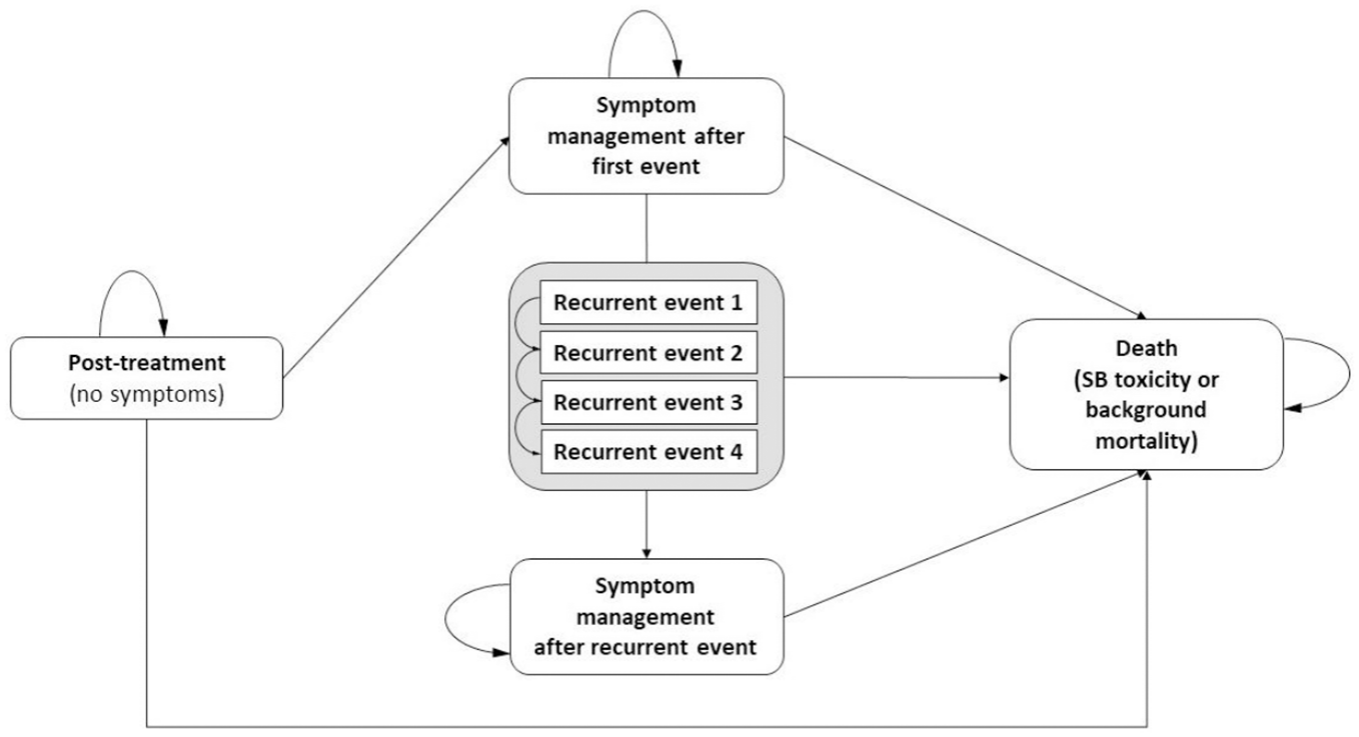
Markov model. SB, Small Bowel.

In this model-based economic evaluation, evidence is drawn from a broad range of sources in the literature (23). In total, 22 different sources were used to populate the model. All sources were selected in careful consideration of the specific context regarding patient population and local context (for example, in the valuation of health outcomes and costs). All sources are listed in detail in the underlying text and/or **Table 1**. The evaluation consists of a Markov model representing the natural history of SB toxicity after RT in survivors of RC (**Figure 1**). Markov models are routinely used in disease courses which imply events over time (42). Additionally, so called Markov cycle trees were developed to simulate treatment pathways in the occurrence of an event (**Supplementary Material Data Sheet 1**). The Markov cycle tree approach is suitable for complex, aggregated pathways which imply temporary loss of QoL and one-time costs which is the case (42, 43).

**Table 1:** Description of parameters, base case value and distribution.

Input parameters	Base-case value	Probability distribution	Source
General setup			
Probability of SB toxicity (converted to half-yearly transition probabilities), % …			
…in IMRT/IGRT			
Grade 1	5.088	###^+^	Calculated from Engels et al. (24) and Engels et al. (25)
Grade 2	1.594	###^+^	
Grade 3	0.658	###^+^	
Grade 4	0.216	###^+^	
…in IMRT			
Grade 1	7.491	###^+^	Calculated from Engels et al. (24) and Engels et al. (25)
Grade 2	2.346	###^+^	
Grade 3	0.969	###^+^	
Grade 4	0.317	###^+^	
… in 3D-CRT			
Grade 1	11.166	###^+^	Calculated from Engels et al. (24) and Engels et al. (25)
Grade 2	3.497	###^+^	
Grade 3	1.444	###^+^	
Grade 4	0.473	###^+^	
Treatments: proportion of patients and treatment efficacy (converted to half-yearly transition probabilities), %			
Prob. of symptom management after loperamide	92.80	β (α = 41.00; β = 22.00)	Chapaux et al. (26)
Prop. of patients treated with AB	44.44	β (α = 32.00; β = 40.00)	Gadhok et al. (27)
Prop. of patients treated with BAS	55.56	β (α = 40.00; β = 32.00)	Gadhok et al. (27)
Prob. of symptom management after AB	41.85	β (α = 15.00; β = 17.00)	Gadhok et al. (27)
Prob. of symptom management after BAS	30.87	β (α = 14.00; β = 26.00)	Gadhok et al. (27)
Prob. of surgery after hospitalization	22.80	β (α = 61.00; β = 90.00)	Pricolo et al. (28)
Prob. of sup. treat. after hospitalization	77.20	β (α = 90.00; β = 61.00)	Pricolo et al. (28)
Prob. of symptom management after surgery	96.22	β (α = 32.64; β = 15.36)	Boland et al. (29)
Prob. of death due to SB toxicity after surgery	3.783	β (α = 32.64; β = 15.36)	Boland et al. (29)
Prob. of symptom management after sup. treat.	99.16	β (α = 167,775; β = 2,845)	Matsushima et al. (30)
Prob. of death due to SB toxicity after sup. treat.	0.837	β (α = 2,845; β = 167,775)	Matsushima et al. (30)
Risk of recurrences (converted to half-yearly transition probabilities), %			
Recurrence 1 after surgery	1.181	β (α = 8.277; β = 65.62)	Behman et al. (31)
Recurrence 1 after sup. treat.	2.106	β (α = 42.61; β = 179.7)	Behman et al. (31)
Recurrence 2 after surgery	2.124	β (α = 1.174; β = 4.904)	Behman et al. (31)
Recurrence 2 after sup. treat.	4.852	β (α = 63.86; β = 99.09)	Behman et al. (31)
Recurrence 3 after surgery	1.965	β (α = 0.062; β = 0.283)	Behman et al. (31)
Recurrence 3 after sup. treat.	6.332	β (α = 41.77; β = 45.23)	Behman et al. (31)
Recurrence 4 after surgery	3.678	β (α = 63.86; β = 99.09)	Behman et al. (31)
Recurrence 4 after sup. treat.	7.726	β (α = 0.057; β = 0.124)	Behman et al. (31)
Mortality			
Background mortality	*Life table, age-dependent*	###	StatBel (32)
Cost parameters: direct costs (pharmaceuticals and hospitalization costs), €			
Unit cost loperamide	0.043	γ (κ = 100.0; θ = 4.312^-4^)	FAMHP (33)
Unit cost AB	0.011	γ (κ = 100.0; θ = 12.70^-4^)	FAMHP (33)
Unit cost BAS	0.127	γ (κ = 100.0; θ = 1.082^-4^)	FAMHP (33)
Unit cost hospitalization for surgery	11,495.840*	γ (κ = 100.0; θ = 115.0)	FBS Health (34)
Unit cost hospitalization for sup. treat.	4,760.970*	γ (κ = 100.0; θ = 47.61)	FBS Health (34)
Cost parameters: indirect costs (productivity costs)			
Work activity rate in Belgium, %	75.10	β (α = 75.10; β = 24.90)	StatBel (35)
Daily wage in Belgium, €	250.118*	γ (κ = 100.0; θ = 2.501)	StatBel (36)
Annual wage in Belgium, €	55,025.900*	γ (κ = 100.0; θ = 550.3)	StatBel (36)
Sick leave days after first/recurrent event	14.550	log-normal (μ log = 2.678)	Khalili et al. (37)
Sick leave days after symptom management	9.650	log-normal (μ log = 2.677)	Khalili et al. (37)
Factor additional sick leave after hospitalization	0.500	log-normal (μ log = -0.693)	*Assumption: expert opinion* ^±^
Utility parameters: Health state utility values^○○^, QALYs			
Utility “post-treatment” state	age-dependent	###	Calculated from De Gendt et al. (9), Ameri et al. (38) and Van Wilder (39)
Relative disutility “pharmaceutical treatment”	-24.7%	###	Calculated from Worbes-Cerezo et al. (40)
Relative disutility “supportive treatment”	-32.3%	###	
Relative disutility “supportive treatment”	-38.7%	###	

+ Random sampling was based on beta distribution for the NTCP as calculated from Engels et al. (25).

* All prices were converted to current pricing (2022, EURO) via the ‘CCEMG – EPPI-Centre Cost Converter’ (41).

± A clinician (MDR) was closely involved in the development of the model and was consulted to estimate additional sick leave days after hospitalization for surgery or supportive treatment.

**^○○^** Cancer-specific EORTC QLQ-C30 values from a Belgian (Flemish) study were mapped on the generic EQ-5D-5L via a mapping algorithm designed specifically for colorectal cancer patients (38). After mapping, relative disutility (compared to healthy Flemish population) was calculated and applied to utility values for the Belgian population. More details on utility calculations are presented in **Supplementary Material Data Sheet 2**.

3D-CRT, Three-Dimensional Conformal Radiation Therapy; AB, Antibiotics; BAS, Bile Acid Sequestrants; IMRT, Intensity Modulated Radiation Therapy; IMRT/IGRT, Intensity Modulated Radiation Therapy – Image Guided Radiation Therapy; NTCP, Normal Tissue Complication Probability; Prob, Probability; Prop, Proportion; SB, Small Bowel; sup. treat., supportive treatment.

### 2.3 Rationale and description of the model

All patients enter the model in the symptom free ‘post-treatment’ state. During each half-yearly cycle, patients may experience grade 1-4 SB toxicity, implicitly transitioning through a decision tree, and resulting in either symptom management (‘symptom management after first event’ state) or death (‘death’ state). The probability of such a SB event was retrieved from a Belgian study which reported late toxicity in 108 patients who were preoperatively treated with Intensity Modulated Radiation Therapy – Image Guided Radiation Therapy (IMRT/IGRT) for locally advanced RC between October 2005 and January 2010 (24). Patients received either a dose of 46Gy or 55.2Gy in daily fractions of 2Gy or 2.4Gy (simultaneous integrated boost of 0.4Gy), respectively (24). The SB volume receiving more than 15Gy was minimized (V15_SB_<150ml) (24). The SB event probabilities were computed by converting trial data rates to probabilities as proposed by Briggs et al. (44). As detailed point estimates on late toxicity over time are lacking, it was assumed that this probability rate remained constant in the first 5 years after treatment yet declined by 25% during each year afterwards. According to the late toxicity criteria developed by the RTOG/EORTC (7) patients with grade 1 and 2 experience mild to moderate diarrhea requiring only pharmaceuticals (antidiarrheal agents, bile acid sequestrants and/or antibiotics) to control symptoms (45). Recovering from grade 1 and 2 was presumed evident, although recurrence could occur in subsequent cycles requiring more invasive treatment. In contrast, grade 3 and 4 involves obstruction, bleeding and/or perforation necessitating hospitalization for supportive (non-surgical) treatment or surgery (45, 46). The probability of needing supportive treatment or surgery was based on a study exploring a non-operative approach in SB obstruction (28).

After a first event, patients may either remain in the ‘symptom management’ state or transition to the ‘recurrent event’ state. Recurrence results in obstruction or perforation and subsequently leads to hospitalization. The probability of recurrence depends on the treatment trajectory during the first/previous event, i.e. recurrences become less likely after surgery compared to non-operative treatment (31). Recurrent events are tunnel states, meaning that patients cannot remain in the ‘recurrent event’ state (47). At the end of the cycle in which the patient encountered a recurrence, he/she makes a transition to [1] ‘recurrent event *x*’ [2], ‘symptom management after recurrent event’ or [3] ‘death’. The effectiveness of these treatments was retrieved from several publications reporting on gastrointestinal disorders in patients with RC (26, 27, 29).

At each cycle, patients might die either from SB toxicity or death from other causes. Death due to SB toxicity was one of the endpoints in decision trees which included the hospitalization pathway. Probability of death was based on research in patients with adhesive SB obstruction and radiation-induced post-resection SB syndrome (29, 30). Death from other causes, the background mortality, was taken into account and based on Belgian age-specific mortality rates in 2020 (32). These probabilities are summarized in **Table 1**.

### 2.4 Comparators

In the current study, Three-Dimensional Conformal Radiation Therapy (3D-CRT) was compared to Intensity Modulated Radiation Therapy (IMRT) and IMRT/IGRT. Research demonstrated a declining trend in normal tissue complication probability (NTCP) in more advanced radiation techniques (i.e. 39.5% in 3D-CRT, 28.5% in IMRT and 18.0% in IMRT/IGRT) as non-tumor tissue is more preserved from radiation. This NTCP-model was confirmed in a clinical phase II study (25). These NTCPs were used as ratios (IMRT/IGRT: 1.0, IMRT: 1.48, 3D-CRT: 2.19) and were applied to the calculated SB toxicity event probabilities from Engels et al. (24). The half-yearly transition probabilities resulting from this computations are presented in **Table 1**. Presumably, this corresponds with lower numbers of SB toxicity events in the two more advanced techniques. Subsequently, less SB events lead to lower costs and higher QoL in these patients.

### 2.5 Valuation of health outcome

In health economics, health outcomes are usually expressed in generic measures of health gain such as QALYs (15). However, the QoL in Belgian survivors of RC was evaluated via the disease-specific EORTC QLQ-C30 questionnaire which do not directly provide QALYs for all health states in the model (9, 15). To overcome this issue, a mapping algorithm was used to convert EORTC QLQ-C30 scores to QALYs (38, 48). Relative disutility was calculated and applied to Belgian age-specific EQ-5D-5L population scores to account for the impact of aging on QoL (39). Utility values were for patients in the ‘Post-treatment’ state and ‘Symptom management after first/recurrent event’ yet decreased for one cycle length during transition. This temporary reduction in QoL was calculated by applying different relative disutilities according to the treatment pathway (pharmaceutical, supportive treatment or surgery). Since no specific utilities were available for these specific treatment pathways in patients with RC, proxies were used based on utility values in patients with Crohn’s disease who experienced similar events (40). Utilities are presented in **Table 1** (for details on utility calculations, see **Supplementary Material Data Sheet 2**).

### 2.6 Valuation of costs, currency and conversion

Costs were estimated using a micro-costing approach allowing more precise calculations based on resource use and unit costs (49). Resource use was estimated using clinical practice guidelines reported in the literature. The unit costs were mainly derived from national unit cost databases. Costs are summarized in **Table 1**. All costs were converted to 2022 Belgian euros via the CCEMPG-EPPI-Centre Cost Converter, version 1.6 (41).

### 2.7 Time horizon and discount rate

A life-time horizon up until the age of 100 was chosen (i.e. patients were modelled until 100 years old or premature death from all causes). Cost and utilities were discounted at a rate of 3.0% and 1.5%, respectively, according to Belgian guidelines (50).

### 2.8 Analytical methods

For all analyses, expected costs and QALYs due to SB toxicity were calculated for the theoretical cohort consisting of patients exposed to RT for RC (i.e. IMRT/IGRT, IMRT or 3D-CRT). In contrast to traditional HE models, results were presented as (mean) incremental costs and (mean) incremental utility rather than (negative) incremental cost-effectiveness ratios (ICERs). However, it seems justified to present the results as such since it allows exploring the benefits by assessing incremental costs and utility separately.

In base case analyses, results were based on most likely assumptions and input parameters extracted from various sources (51). In addition, in a deterministic scenario analysis, the cumulative number of hospitalizations per 100 patients was modelled for cohorts of 30-, 50-, 70-, and 90-year old patients.

To assess parameter uncertainty in the model, base case assumptions and input parameters were varied consistent to HE modelling guidelines (51). The most influential parameters were identified by deterministic one-way sensitivity analyses. In these analyses, the input parameters were adjusted separately by setting each parameter to 70% and 130% relative to the base case value which is a conventional HE method in such analysis. Results were presented in Tornado diagrams (52). In probabilistic sensitivity analyses (PSA) 1,000 Monte-Carlo simulations were performed according to the distributions presented in **Table 1** (53). The results of PSA were presented in cost-effectiveness planes (54).

## 3 Results

### 3.1 Incremental costs and utility comparing 3D-CRT with IMRT/IGRT and IMRT

The base case analysis showed that the incremental cost of SB toxicity compared to 3D-CRT is -€3,820 and -€1,863 per patient for IMRT/IGRT and IMRT, respectively. The analysis resulted in 0.11 QALYs gained in patients treated with IMRT/IGRT and 0.05 QALYs gained in IMRT patients. When excluding indirect costs from analysis, incremental (direct) costs are -€922 and -€434 for IMRT/IGRT and IMRT, respectively. These results are summarized in **Table 2**.

**Table 2:** Overview of discounted and undiscounted absolute and incremental* costs and QALYs (mean per patient).

	3D-CRT		IMRT/IGRT				IMRT			
	Absolute costs	Absolute QALYs	Absolute costs	Absolute QALYs	Incr. costs	Incr. QALYs	Absolute costs	Absolute QALYs	Incr. costs	Incr. QALYs
Discounted	€11,820	20.40	€7,999	20.50	-€3,820	+0.11	€9,956	20.45	-€1,863	+0.05
Undiscounted	€15,092	24.19	€10,420	24.31	-€4,672	+0.12	€12,857	24.25	-€2,235	+0.06

*Incremental costs, absolute cost IMRT/IGRT or IMRT – absolute cost 3D-CRT.

*Incremental QALYs, absolute QALYs IMRT/IGRT or IMRT – absolute QALYs 3D-CRT.

3D-CRT, Three-Dimensional Conformal Radiation Therapy; Incr, Incremental; IMRT, Intensity Modulated Radiation Therapy; IMRT/IGRT, Intensity Modulated Radiation Therapy – Image Guided Radiation Therapy.

### 3.2 Hospitalizations after RC due to SB toxicity

**Table 3** reflects the cumulative sum of hospitalizations related to SB toxicity after 3D-CRT, IMRT/IGRT and IMRT for 4 homogenous cohorts each modelling 100 patients (30 years, 50 years, 70 years and 90 years at diagnosis). First events and recurrences were taken into account. This analysis resulted in substantial differences between IMRT/IGRT, IMRT and 3D-CRT. For example: SB toxicity will result in 36 hospitalizations in 100 patients with RC (30 year-olds) in the first 10 years following 3D-CRT whereas there would be 12 and 7 hospitalizations less, respectively, if IMRT/IGRT or IMRT were preferred over 3D-CRT in the same cohort.

**Table 3:** Cumulative sum of hospitalizations (first events and recurrences) per cohort of 100 patients (absolute numbers).

	Time horizon (years after treatment)																				
	10y			20y			30y			40y			50y			60y			70y		
Age at diagnosis	3D-CRT	IMRT	IMRT/IGRT	3D-CRT	IMRT	IMRT/IGRT	3D-CRT	IMRT	IMRT/IGRT	3D-CRT	IMRT	IMRT/IGRT	3D-CRT	IMRT	IMRT/IGRT	3D-CRT	IMRT	IMRT/IGRT	3D-CRT	IMRT	IMRT/IGRT
30y	36	31	24	61	53	44	77	68	56	86	77	64	92	82	68	94	84	70	94	84	70
50y	36	30	24	59	51	42	71	63	52	76	68	56	77	68	57	NA	NA	NA	NA	NA	NA
70y	33	28	22	46	40	33	48	42	35	NA	NA	NA	NA	NA	NA	NA	NA	NA	NA	NA	NA
90y	16	12	10	NA	NA	NA	NA	NA	NA	NA	NA	NA	NA	NA	NA	NA	NA	NA	NA	NA	NA

3D-CRT, Three-Dimensional Conformal Radiation Therapy; IMRT, Intensity Modulated Radiation Therapy; IMRT/IGRT, Intensity Modulated Radiation Therapy – Image Guided Radiation Therapy; y, Years.NA, Not Applicable.

### 3.3 Sensitivity analyses

Deterministic one-way sensitivity analyses revealed that incremental cost mostly depend on probabilities determining the risk of SB toxicity. Furthermore, since a societal perspective is adopted, parameters related to indirect costs were identified as influential for incremental cost (e.g. wages and number of sick leave days). These analyses revealed that incremental utility is mainly affected by the efficacy of treatments (i.e. surgery and supportive treatment) but also -in a much smaller extend- by transition probabilities (e.g. risk of SB toxicity, probability of supportive treatment after hospitalization and probability of recurrences) and relative disutility values. Tornado diagrams are presented in **Supplementary Material Data Sheets 3 and 4**.

**Figure 2:**
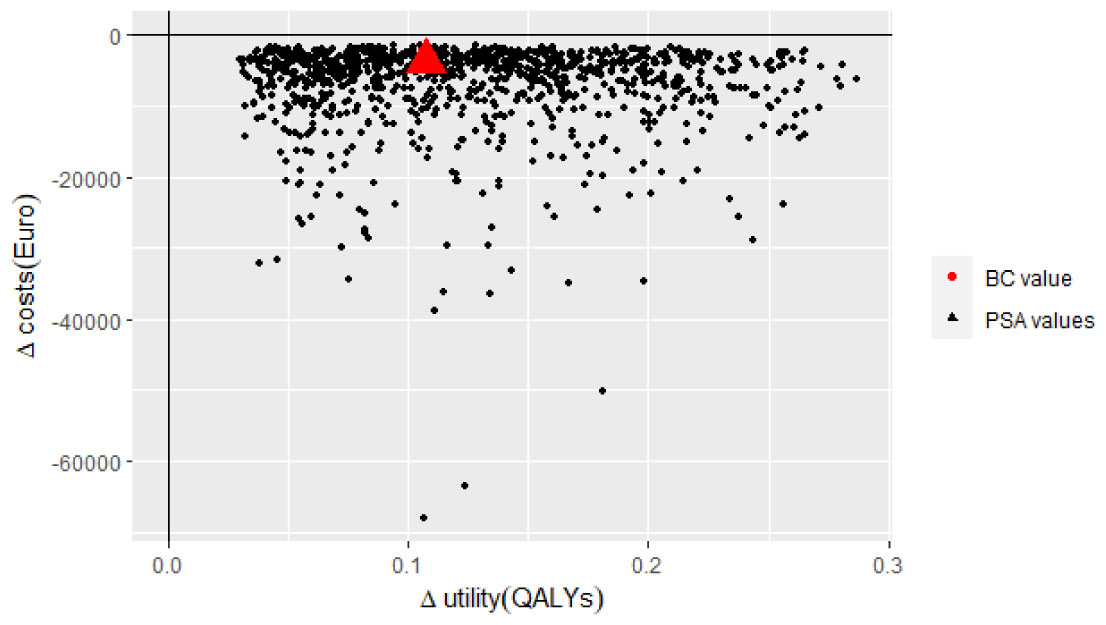
South-east quadrant of the cost-effectiveness plane: IMRT/IGRT vs. 3D-CRT. Representation of results (i.e. incremental cost and utility) representing 1,000 Monte Carlo simulations comparing IMRT/IGRT to 3D-CRT. 3D-CRT, Three-Dimensional Conformal Radiation Therapy; BC, Base Case; IMRT/IGRT, Intensity Modulated Radiation Therapy – Image Guided Radiation Therapy; PSA, Probabilistic Sensitivity Analysis; QALYs, Quality-Adjusted Life Years.

**Figure 3:**
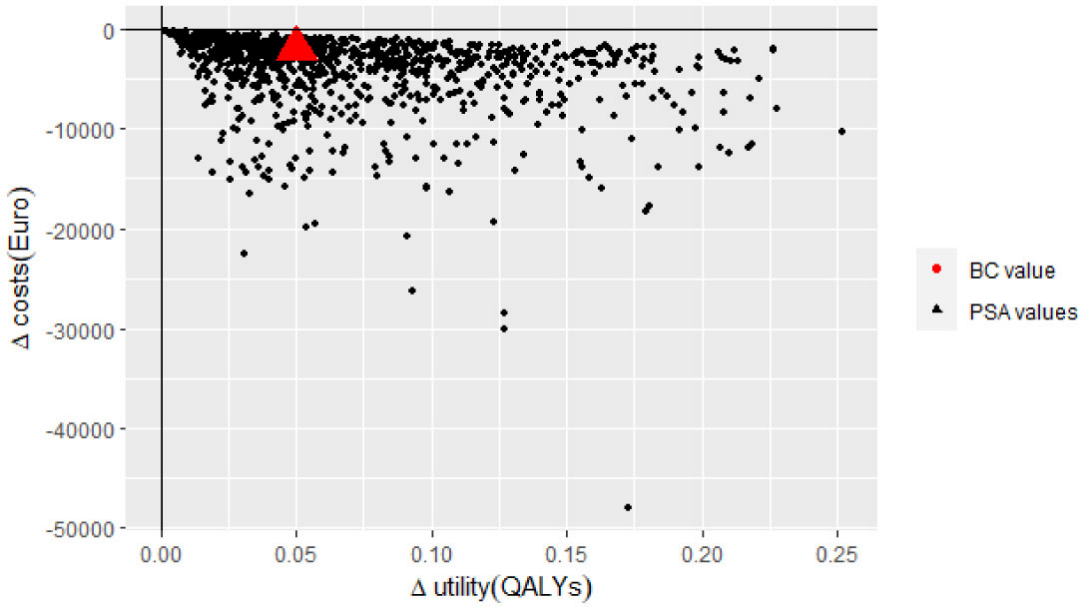
South-east quadrant of the cost-effectiveness plane: IMRT vs. 3D-CRT. Representation of results (i.e. incremental cost and utility) representing 1,000 Monte Carlo simulations comparing IMRT to 3D-CRT. 3D-CRT, Three-Dimensional Conformal Radiation Therapy; BC, Base Case; IMRT, Intensity Modulated Radiation Therapy; PSA, Probabilistic Sensitivity Analysis; QALYs, Quality-Adjusted Life Years.

The probabilistic sensitivity analysis demonstrated that the incremental costs and utility for IMRT/IGRT compared to 3D-CRT varied from -€1,393 to -€67,965 and from +0.03 QALYs to +0.29 QALYs, respectively. The analysis for IMRT vs. 3D-CRT ranged from -€69 to -€47,977 for incremental costs and from +0.002 QALYs to +0.25 QALYs for incremental utility. The expected values are presented in the cost-effectiveness planes in **Figures 2**, **3**.

## 4 Discussion and conclusion

In this study, we assessed the long-term impact of SB toxicity induced by IMRT/IGRT and IMRT and compared it to 3D-CRT. While the introduction of 3D-CRT marked substantial advancements in reducing NTCP compared to the era of two-dimensional RT, the evolution of modern RT (such as IMRT and IMRT/IGRT) has further enabled the treatment team to enhance precision in targeting tumor tissue (55, 56). This allows for more refined dose delivery to the target tissue while minimizing the dose delivery to surrounding tissue and subsequently mitigating the likelihood of complications (55, 56). In effect, our analyses showed that the use of innovative RT improves lifetime QoL in survivors of RC by 0.11 QALYs and 0.05 QALYs by preferring IMRT/IGRT or IMRT over 3D-CRT, respectively. Furthermore, the use of IMRT/IGRT and IMRT results in an incremental saving of €3,820 and €1,863 per patient (i.e. direct and indirect costs resulting from SB toxicity) compared to 3D-CRT. In our study, we estimated radiation-induced SB toxicity admissions in 4 age-specific cohorts each consisting of 100 patients with RC. We made the assumption that the severity of symptoms in grade 3 and 4 toxicity and recurrent events require hospital admission. Birgisson et al. (57) examined late admissions via administrative data. In their cohort of 454 patients who received RT, 157 gastrointestinal-related admissions (i.e. 35 admissions/100 patients) occurred during the follow-up period (max. 11-14 years) (57). This approximates our results of 22 to 32 admissions per 100 70-year old patients during the first 10 years after treatment. Furthermore, one-way sensitivity analysis identified probabilities for SB toxicity as most influential parameters. In our analysis, the NTCP ratios for 3D-CRT, IMRT and IGRT were multiplied by the transition probabilities of SB toxicity to compute the transition probabilities for the three assessed RT techniques (NTCP IGRT: 0.180, 1.00; NTCP IMRT: 0.265, ratio: 1.48; NTPC 3D-CRT: 0.395, ratio: 2.19). We assessed risk reduction and adverse treatment effect probabilities used in other cost-effectiveness studies evaluating 3D-CRT and IMRT for pelvic radiation. Carter et al. (58) employed a risk reduction of 0.67 (range: 0.35-0.92) for late gastrointestinal (GI) toxicity in IMRT patients compared to 3D-CRT patients (58), which corresponds well with the ratio we used in our study (1.48/2.19 = 0.68). Hodges et al. (59) reported GI toxicity (≥G3) probabilities of 0.210 and 0.360 for IMRT and 3D-CRT, respectively, comparable to the probabilities of 0.265 and 0.395 that we used in our analyses (59). It should be noted that one economic evaluation in prostate cancer (60) and a study on toxicity profiles in patients with RC (61) propose remarkable higher late GI toxicity probabilities for IMRT and 3D-CRT. Nevertheless, we believed a more conservative approach towards estimating the NTCP was necessary to avoid overestimation.

Research has demonstrated that IMRT is cost-effective compared to 3D-CRT in anal, prostate, head-and-neck and gynecologic cancers (58–60, 62, 63). However, Sun et al. (64) failed to establish similar results for RC due to comparable short-term benefits and long-term survival for IMRT and 3D-CRT in this population (64). Therefore, the former studies emphasize the importance of taking late toxicity outcomes into account when evaluating cost-effectiveness of RT techniques (62, 63). Nowadays, HE analyses are frequently used in health technology assessments (HTA) to inform decision-makers about cost-effective strategies, especially in reimbursement dossiers (65–67). If innovative strategies are not deemed cost-effective in HTA, decision-makers might be reluctant to provide financial incentives to invest in innovation since financial resources are constrained in healthcare (68–70). Hence, Ng et al. (71) stated that capital investments are the most immediate barrier in the wide implementation of IMR-LINAC technology (71). Research demonstrated that nearly all simulators within the European Cancer Observatory network have 3D capacity. More advanced IMRT and IGRT technology is available in only 69% and 49% of the megavoltage units, respectively (72). Although most high income countries are well equipped, there seems to be room for improvement in terms of access to modern machines which are capable of delivering high precision treatments, especially in Eastern European countries (72). In our previous study, we quantified societal burden of radiation-induced cardiotoxicity in breast cancer survivors. We concluded that the marginal gains in further reducing current mean heart doses are limited (73). Thus, it would be an opportunity to shift the focus to other long-term side effects such as SB toxicity in RC. It is important to highlight the societal perspective we obtained in our analyses. In this perspective all costs and benefits are included regardless of who encountered them. Hence, it would be inappropriate to draw conclusions about investments at the level of hospitals or radiotherapy departments based on our findings (74, 75). Defourny et al. (76) concluded that other methods (such as micro-costing and time-driven-activity-based-costing methods) are more frequently employed when performing HE evaluations from an institutional level (76). Investment decisions at this level require an in-depth analysis of costs and resource use at hospital level which go beyond the scope of the current study (75, 77).

Several limitations should be pointed out. Our model is populated with data from various literature sources. Health economic models build on the available evidence and on structural assumptions (66, 78, 79). First off, although we endeavored to construct a model which represents up to date clinical practice it is possible that some assumptions differ from local protocols and guidelines. For example, the treatment pathways in the occurrence of a SB event (i.e. decision trees) are based on guidelines for the management of symptoms in patients with RC (26, 27, 29, 45, 46). but also on studies addressing similar symptoms of interest in other populations (28, 30, 31). Also, the utility values are conditional to the mapping of EORTC QLQ-C30 scores to QALYs. However, according to Drummond et al. (48) mapping to predict EQ-5D based on EORTC QLQ-C30 scores is appropriate in cancer studies (48). Therefore, the mapping algorithm designed by Ameri et al. (38) was applied to map QLQ-C30 scores from patients with RC to EQ-5D-5L (38). It is important to notice that cost, and cost savings, are subject to local context (77, 80). In effect, unit costs in the current study are retrieved from Belgian sources which makes it important to consider the local context before transferring the results to other healthcare systems. Secondly, as the one-way sensitivity analysis indicated, the results are particularly influenced by the transition probabilities for grade 1-4 SB toxicity. We retrieved data for these calculations from a study that fitted well with the characteristics of our theoretical cohort (24, 25). We challenged our model by using the GI toxicity rates published by Azria et al. (81), which is a study with a larger sample size (n = 281). The patients in this study match those in our theoretical cohort (T3-T4 Nx M0 and T2 Nx distal anterior rectum tumors, receiving RT 50 Gy + capecitabine and oxaliplatin arm). This resulted in similar deterministic point estimates (i.e. incremental cost of -€3,630 and -€1,937 and incremental utility of 0.11 QALYs and 0.06 QALYs, by preferring IMRT/IGRT or IMRT over 3D-CRT, respectively) (81).

In the present study we modelled long-term SB toxicity from 3D-CRT, IMRT and IMRT/IGRT in patients with RC. To the best of our knowledge, HE analyses comparing novel RT technologies and older techniques in which long-term effects are taken into account are not yet performed in these patients. Our findings might therefore be useful to complement cost-effectiveness analyses comparing RT techniques in patients with RC. In decisions regarding investments and reimbursement of novel technologies, it is important to consider late toxicity since there is a large potential of saving costs and improving QoL on the long-term.

## Data availability statement

Publicly available datasets were analyzed in this study. This data can be found here: https://belgian-cancer-registry.shinyapps.io/data_app/ (Belgian Cancer Registry- Annual tables: Absolute numbers 2019) https://statbel.fgov.be/en/themes/population/mortality-life-expectancy-and-causes-death/life-expectancy-and-life-tables#figures (Belgian statistical office - Life expectancy and life tables: Life tables, yearly, in exact age) https://www.bcfi.be/nl/start (Belgian federal agency for medicines and health product -Belgian formularium for pharmaceutical information) https://tct.fgov.be/webetct/etct-web/ (Belgian federal public service for health - Technical unit for data processing regarding inpatient care) https://statbel.fgov.be/en/themes/work-training/labour-market/employment-and-unemployment#figures (Belgian statistical office - Employment and unemployment: Annual labour market indicators with regard to activity, sex and age (population aged 15 to 64) and region of residence) https://statbel.fgov.be/en/themes/work-training/wages-and-labourcost/overview-belgian-wages-and-salaries#figures (Belgian statistical office - An overview of Belgian wages and salaries: An overview of Belgian wages and salaries 1999-2019).

## Ethics statement

The studies involving humans were approved by the ethical committee at Universitair Ziekenhuis Brussel. The studies were conducted in accordance with the local legislation and institutional requirements. Written informed consent for participation was not required from the participants or the participants’ legal guardians/ next of kin in accordance with the national legislation and institutional requirements.

## Author contributions

EK: Conceptualization, Data curation, Formal analysis, Methodology, Project administration, Software, Supervision, Validation, Visualization, Writing – original draft, Writing – review & editing. RP: Conceptualization, Methodology, Writing – review & editing. S-LB: Writing – review & editing. JDM: Funding acquisition, Writing – review & editing. KB: Conceptualization, Funding acquisition, Writing – review & editing. MDR: Conceptualization, Funding acquisition, Methodology, Supervision, Writing – review & editing. KP: Conceptualization, Data curation, Formal analysis, Funding acquisition, Methodology, Software, Supervision, Validation, Visualization, Writing – review & editing.
