# Supplementary material for: Quantifying societal burden of radiation-induced small bowel toxicity in patients with rectal cancer
Source: Front Oncol. 2024 Jul 8;14:1340081. doi: 10.3389/fonc.2024.1340081 (PMC11260702; doi:10.3389/fonc.2024.1340081)

frontiers

Supplementary Material A: Markov cycle trees

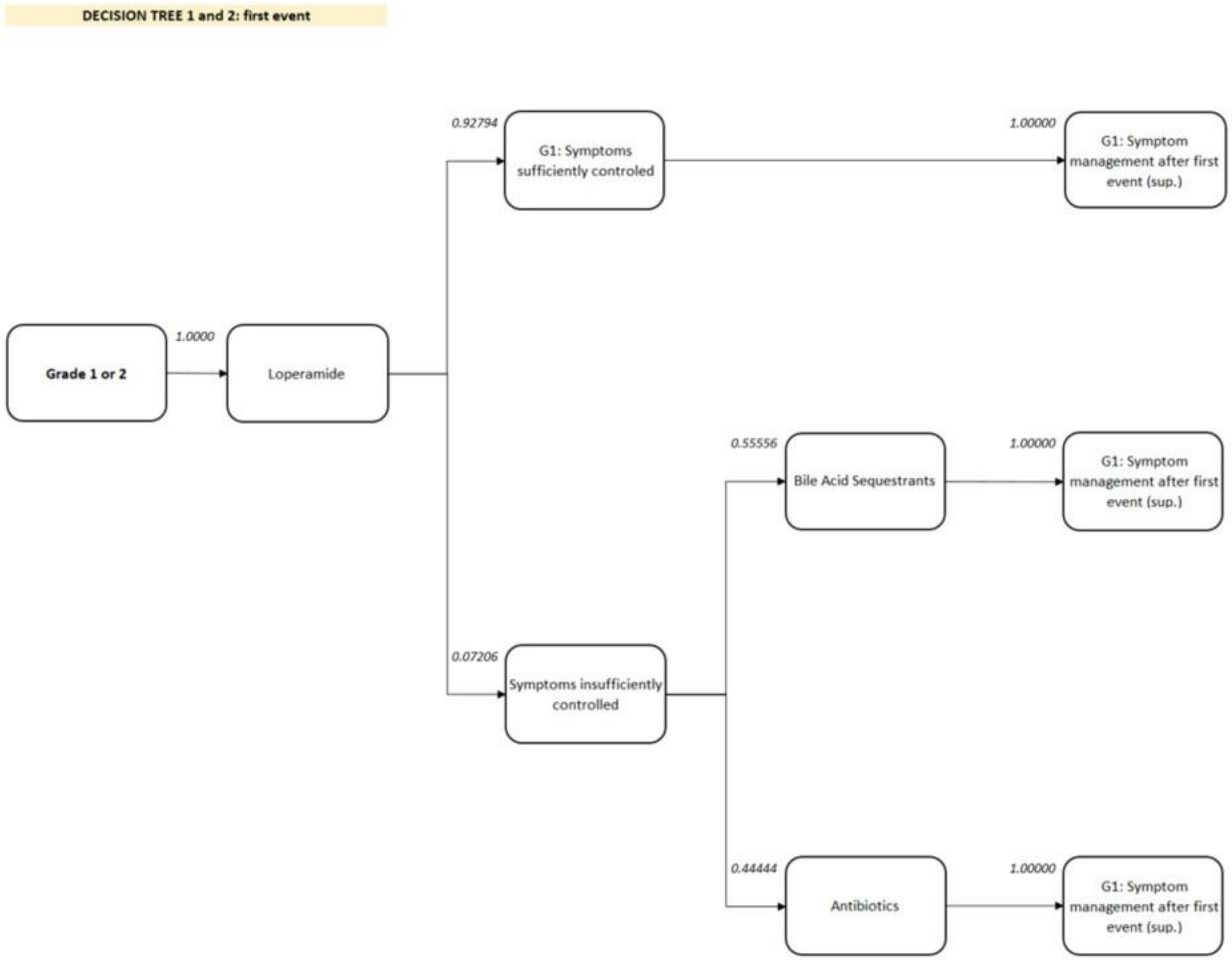

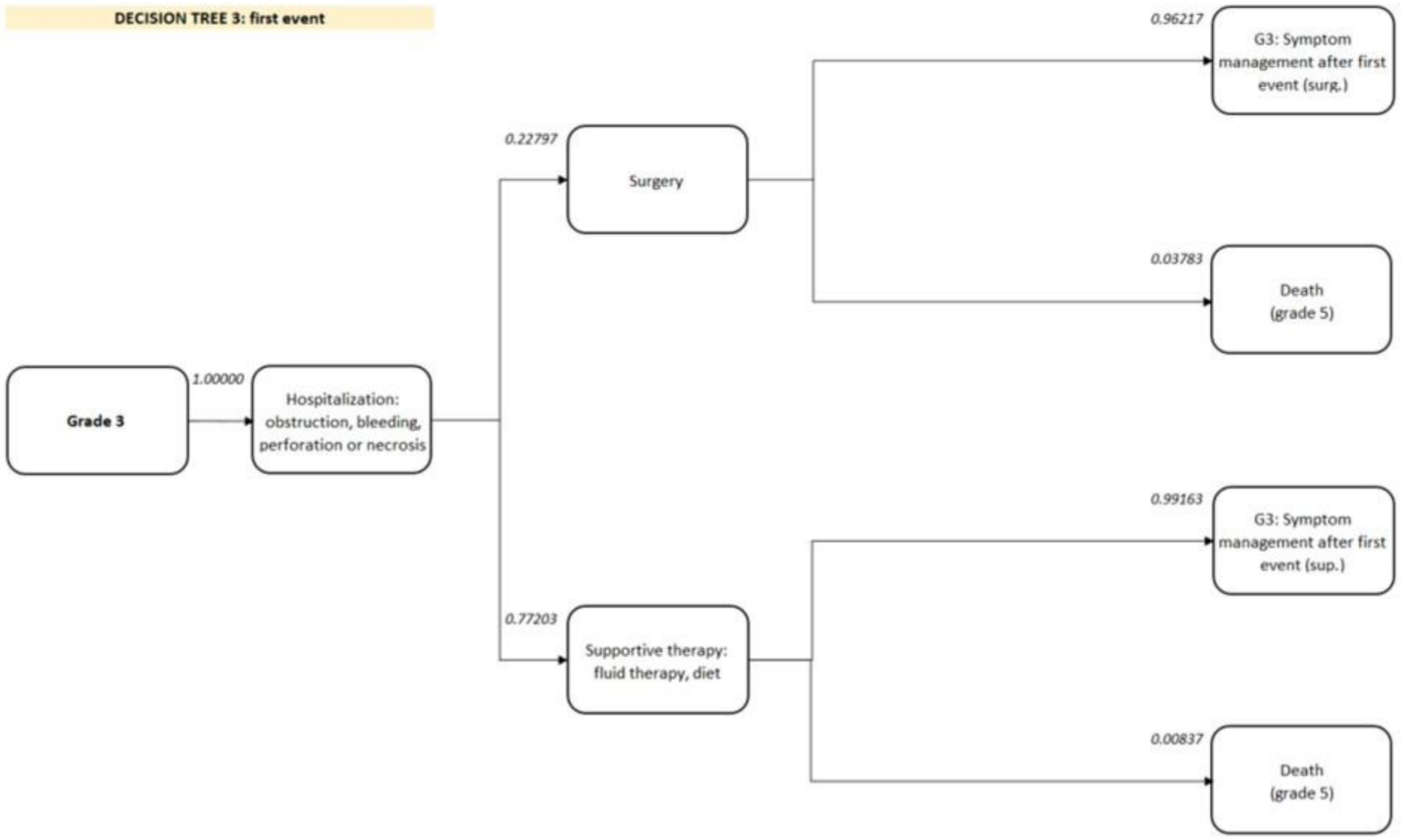
DECISION TREE 3: first event
Grade 3
1.00000
Hospitalization: obstruction, bleeding, perforation or necrosis
0.22797
Surgery
0.77203
Supportive therapy: fluid therapy, diet
0.96217
G3: Symptom management after first event (surg.)
0.03783
Death (grade 5)
0.99163
G3: Symptom management after first event (sup.)
0.00837
Death (grade 5)

DECISION TREE 4: first event

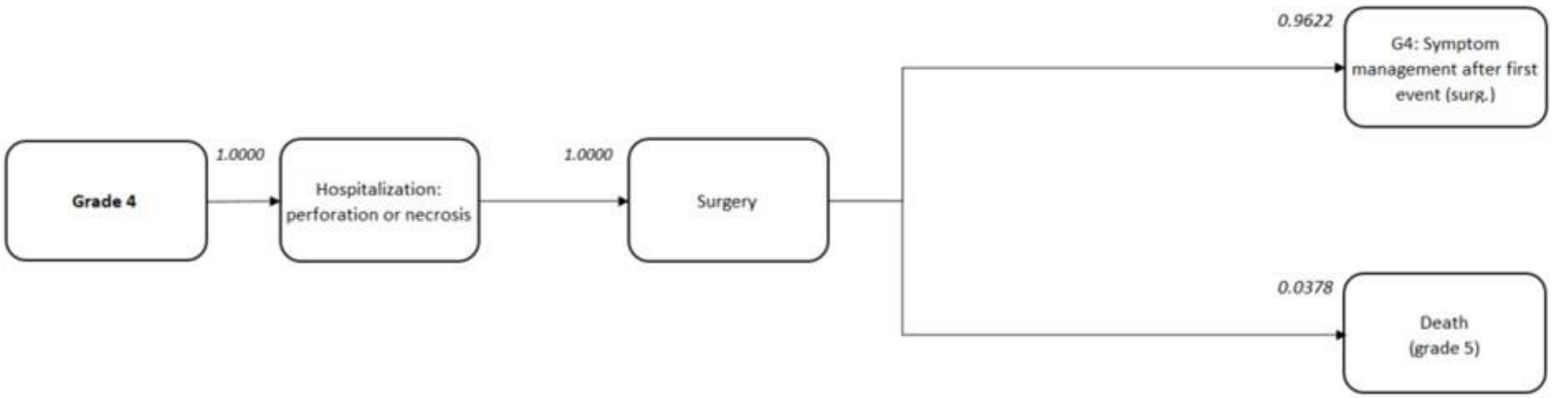

DECISION TREE 5, 6 and 7: recurrent event 1, 2 or 3

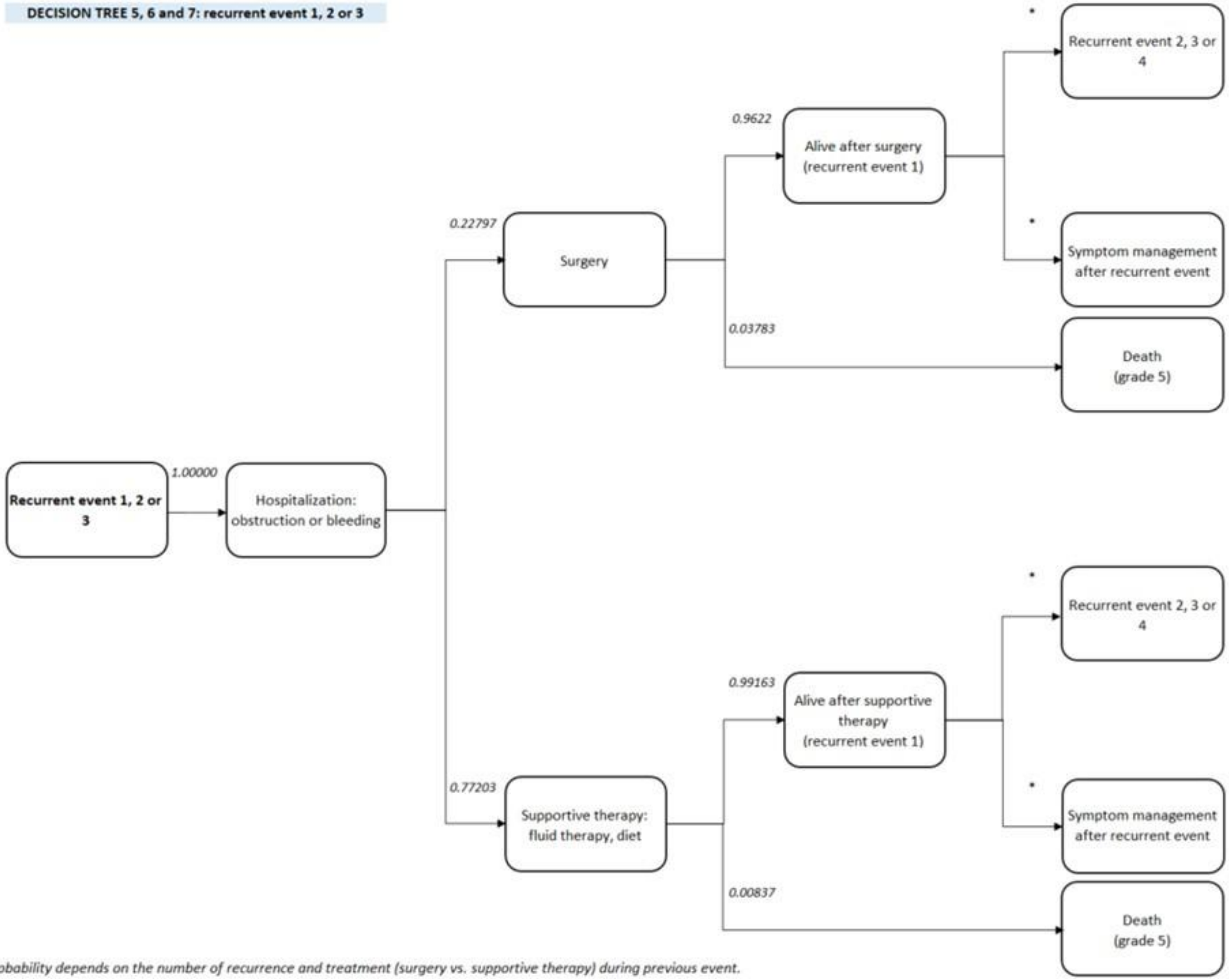

** Probability depends on the number of recurrence and treatment (surgery vs. supportive therapy) during previous event.*

DECISION TREE 8: recurrent event 4

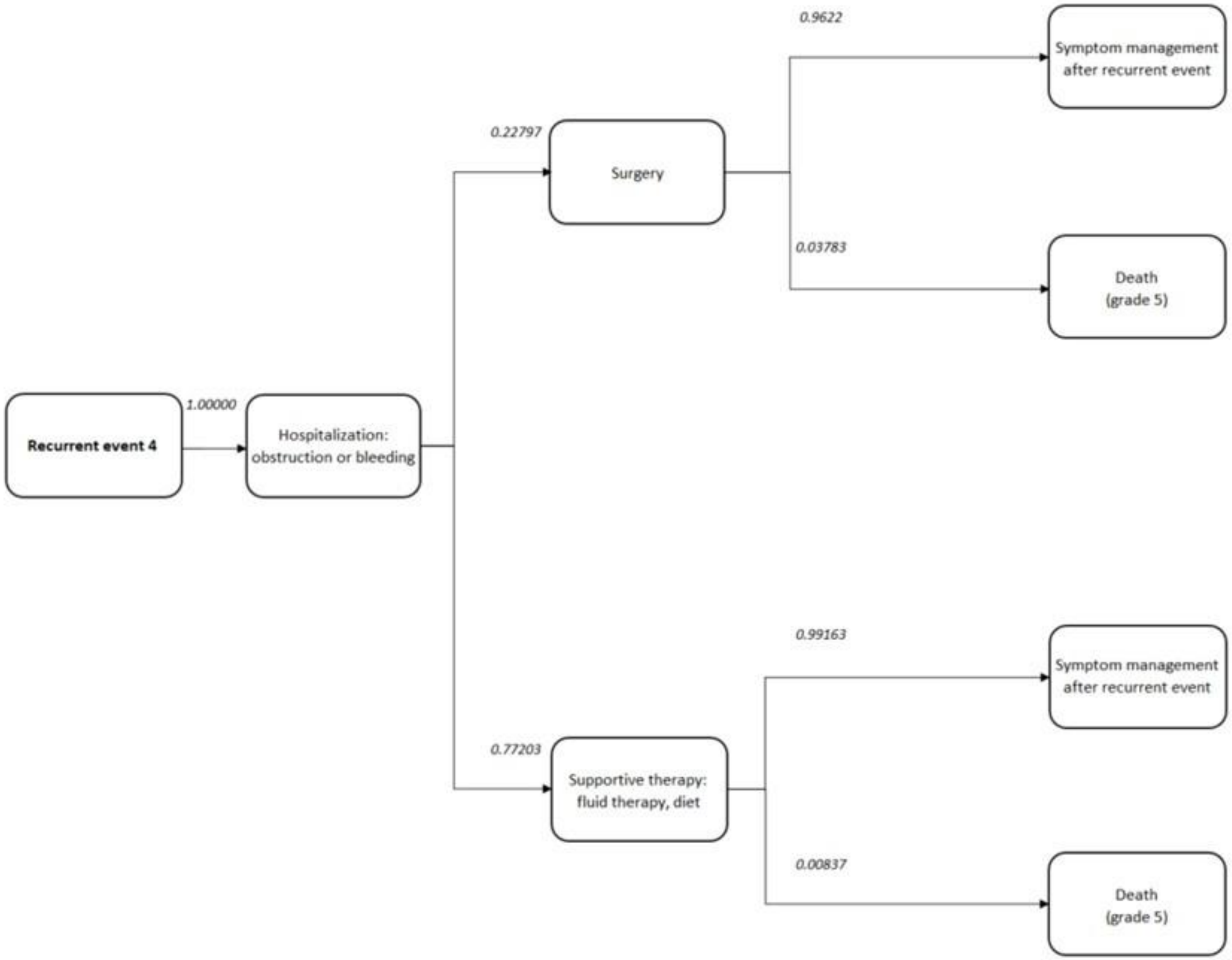

Supplement: Supplementary file 1 [file DataSheet_1.pdf]
