# Supplementary material for: Quantifying societal burden of radiation-induced small bowel toxicity in patients with rectal cancer
Source: Front Oncol. 2024 Jul 8;14:1340081. doi: 10.3389/fonc.2024.1340081 (PMC11260702; doi:10.3389/fonc.2024.1340081)
Supplement: Supplementary file 2 [file DataSheet_2.pdf]

Supplementary Material B: Absolute and relative disutility calculation

1. <u>Calculation of utility value for patients in "post-treatment" state</u>

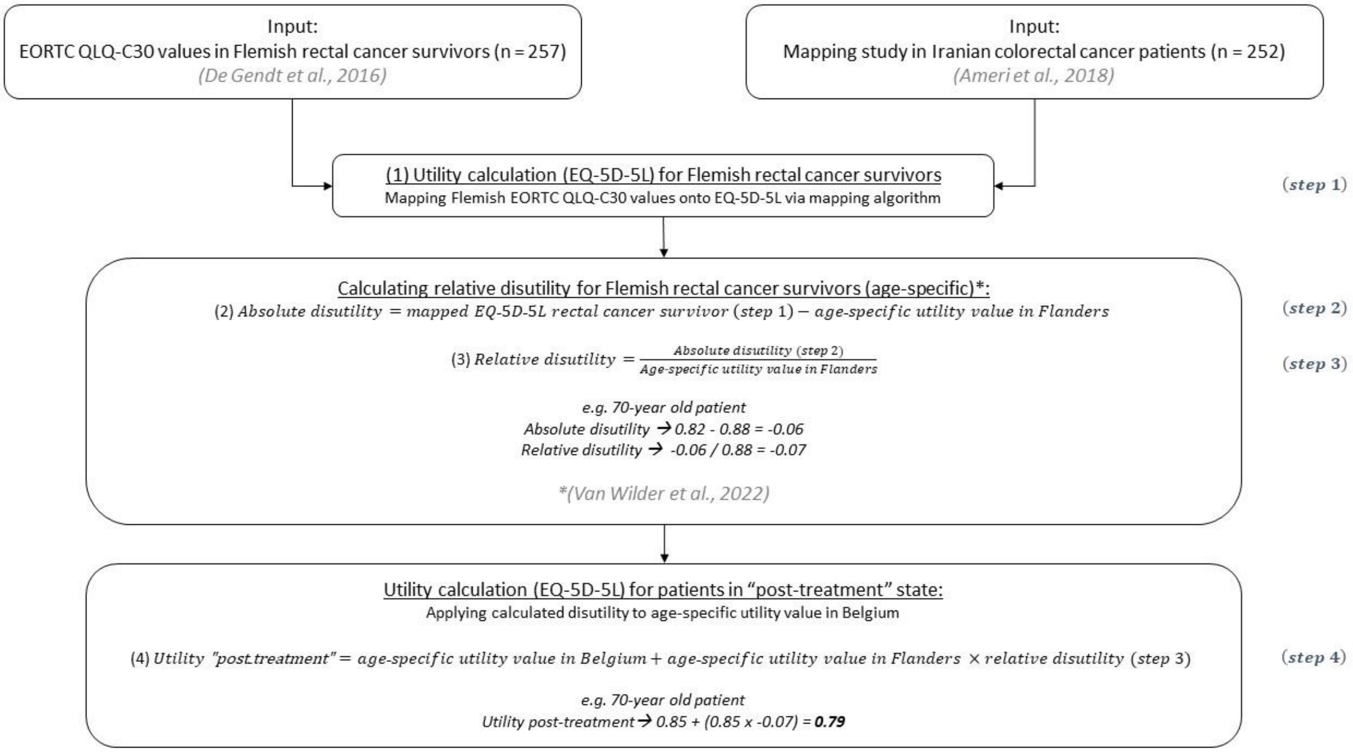

2. Calculation of utility value for patients who transition to "Symptom management" state (differentiation between different treatment pathways, i.e. medication, supportive treatment and surgery)

Input:
British study on utility values (EQ-5D-5L) for different treatments and adverse effects in Crohn's disease via time-trade-off (n = 100)
(5) Determine which treatments and adverse effects in Crohn's disease correspond best with rectal cancer survivors, based on description provided by the authors
(Worbes-Cerezo et al., 2019)

$(step\ 5)$

Calculating absolute disutility for different treatments and adverse effects:

(6) $Absolute\ disutility = utility\ value\ selected\ treatments\ /\ adverse\ effect\ in\ Crohn's\ disease\ (step\ 5) - index\ utility\ for\ Crohn's\ disease$

$(step\ 6)$

Abs. disutility "moderate/severe Crohn" (= transition to "Symptom management" state via pharmaceutical treatment pathway)→ 0.70 – 0.93 = -0.23
Abs. disutility "serious infection" (= transition to "Symptom management" state via hospitalization, supportive treatment)→ 0.63 – 0.93 = -0.30
Abs. disutility "prolonged ileus/bowel obstruction" (= transition to "Symptom management" state via hospitalization, surgery)→ 0.57 – 0.93 = -0.36

Calculating relative disutility for different treatments and adverse effects:

(7) $Relative\ disutility = \frac{Absolute\ disutility\ (step\ 6)}{Index\ utility\ value\ for\ Crohn's\ disease}$

$(step\ 7)$

Rel. disutility "moderate/severe Crohn" (= transition to "Symptom management" state via pharmaceutical treatment pathway) = -0.25
Rel. disutility "serious infection" (= transition to "Symptom management" state via hospitalization, supportive treatment) = -0.32
Rel. disutility "prolonged ileus/bowel obstruction" (= transition to "Symptom management" state via hospitalization, surgery) = -0.39

Utility calculation (EQ-5D-5L) for patients who transition to "Symptom management":
Applying calculated disutility to calculated age-specific utility value for patients in the 'post-treatment' state *(step 4)*

(8) $Utility\ \text{"Symptom management"} = age\text{-}specific\ utility\ value\ post\text{-}treatment\ state + age\text{-}specific\ utility\ value\ post\text{-}treatment\ stage \times relative\ disutility\ (step\ 7)$

$(step\ 8)$

e.g. 70-year old patient, transition to symptom management state via pharmaceutical treatment pathway
Utility Symptom management→ 0.79 + (0.79 x -0.25) = ***0.59***

References

Ameri, H., Yousefi, M., Yaseri, M., Nahvijou, A., Arab, M., & Akbari Sari, A. (2018). Mapping the cancer-specific QLQ-C30 onto the generic EQ-5D-5L and SF-6D in colorectal cancer patients. *Expert Review of Pharmacoeconomics and Outcomes Research*, *19*(1), 89–96. https://doi.org/10.1080/14737167.2018.1517046

De Gendt C, De Coster G, Vandendael T, et al. *Onderzoek Naar Levenskwaliteit Bij Colorectale (Ex-)Kankerpatiënten: Basisrapport [Study on Quality of Life in (Ex-) Colorectal Cancer Patients: Primary Report]*. Brussels, Belgium; 2016. https://kankerregister.org/Quality of Life.

Van Wilder, L., Charafeddine, R., Beutels, P., Bruyndonckx, R., Cleemput, I., Demarest, S., De Smedt, D., Hens, N., Scohy, A., Speybroeck, N., Van der Heyden, J., Yokota, R. T. C., Van Oyen, H., Bilcke, J., & Devleesschauwer, B. (2022). Belgian population norms for the EQ-5D-5L, 2018. *Quality of Life Research*, *31*(2), 527–537. https://doi.org/10.1007/s11136-021-02971-6

Worbes-Cerezo, M., Nafees, B., Lloyd, A., Gallop, K., Ladha, I., & Kerr, C. (2019). Disutility study for adult patients with moderate to severe Crohn's disease. *Journal of Health Economics and Outcomes Research*, *6*(2), 47–60. https://doi.org/10.36469/9685
