# Supplementary material for: Quantifying societal burden of radiation-induced small bowel toxicity in patients with rectal cancer
Source: Front Oncol. 2024 Jul 8;14:1340081. doi: 10.3389/fonc.2024.1340081 (PMC11260702; doi:10.3389/fonc.2024.1340081)
Supplement: Supplementary file 3 [file DataSheet_3.pdf]

Supplementary Material C: Tornado diagrams – One way sensitivity analysis (incremental costs)

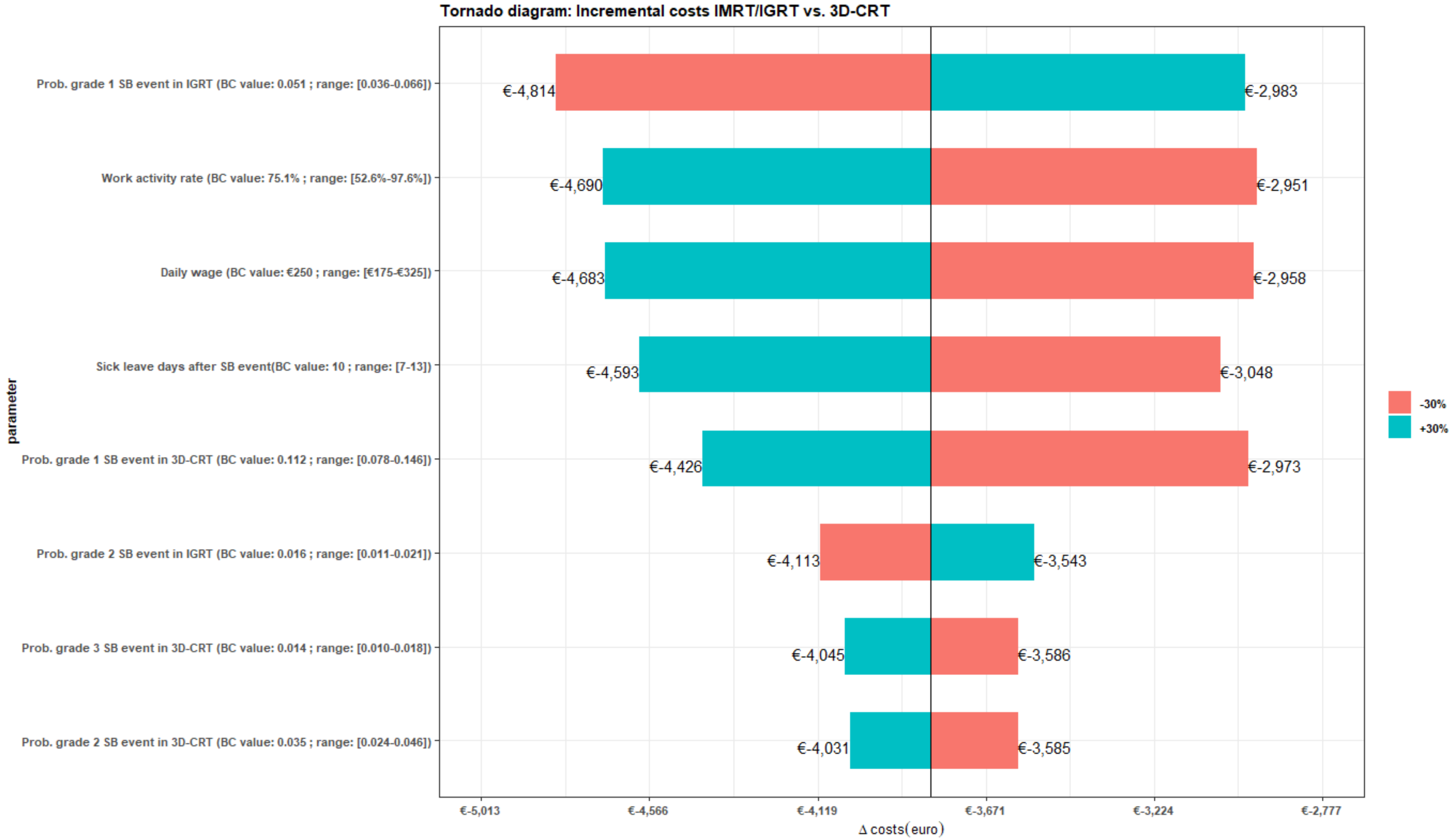

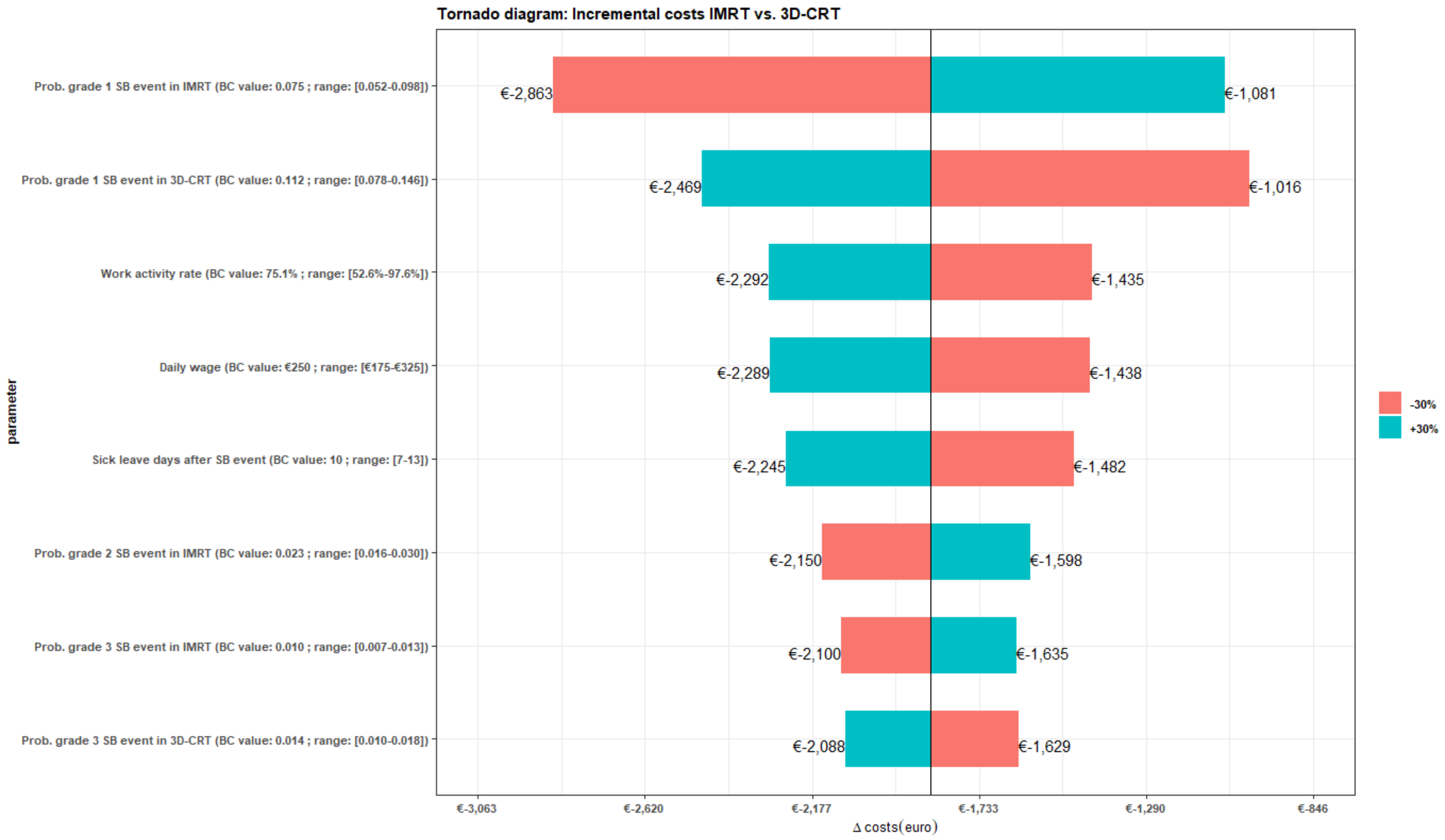
Tornado diagram: Incremental costs IMRT vs. 3D-CRT
parameter
Prob. grade 1 SB event in IMRT (BC value: 0.075 ; range: [0.052-0.098])
Prob. grade 1 SB event in 3D-CRT (BC value: 0.112 ; range: [0.078-0.146])
Work activity rate (BC value: 75.1% ; range: [52.6%-97.6%])
Daily wage (BC value: €250 ; range: [€175-€325])
Sick leave days after SB event (BC value: 10 ; range: [7-13])
Prob. grade 2 SB event in IMRT (BC value: 0.023 ; range: [0.016-0.030])
Prob. grade 3 SB event in IMRT (BC value: 0.010 ; range: [0.007-0.013])
Prob. grade 3 SB event in 3D-CRT (BC value: 0.014 ; range: [0.010-0.018])
€-2,863
€-1,081
€-2,469
€-1,016
€-2,292
€-1,435
€-2,289
€-1,438
€-2,245
€-1,482
€-2,150
€-1,598
€-2,100
€-1,635
€-2,088
€-1,629
€-3,063
€-2,620
€-2,177
€-1,733
€-1,290
€-846
Δ costs(euro)
-30%
+30%
