# Supplementary material for: Quantifying societal burden of radiation-induced small bowel toxicity in patients with rectal cancer
Source: Front Oncol. 2024 Jul 8;14:1340081. doi: 10.3389/fonc.2024.1340081 (PMC11260702; doi:10.3389/fonc.2024.1340081)
Supplement: Supplementary file 4 [file DataSheet_4.pdf]

Supplementary Material D: Tornado diagrams – One way sensitivity analysis (incremental utility)

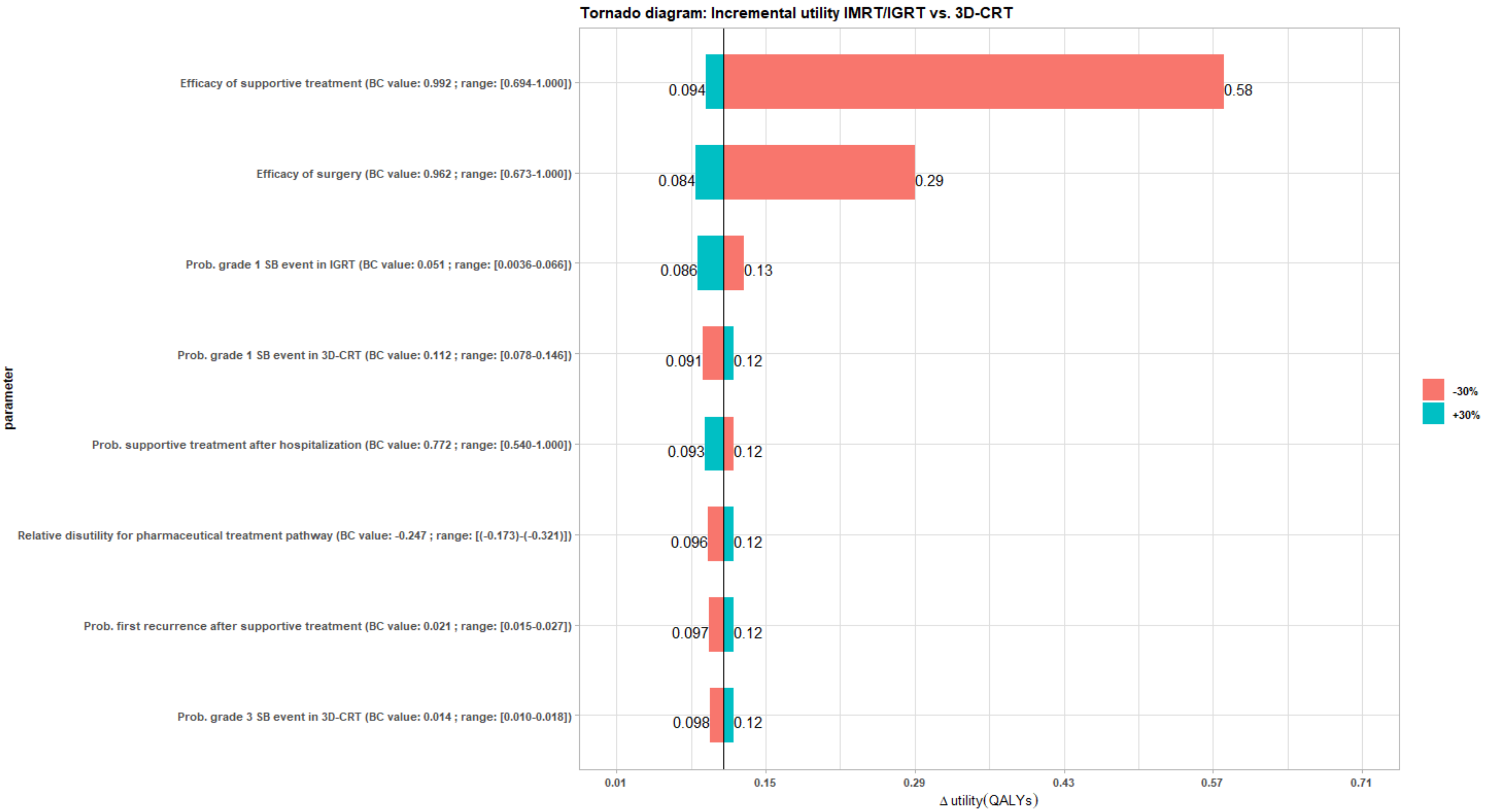

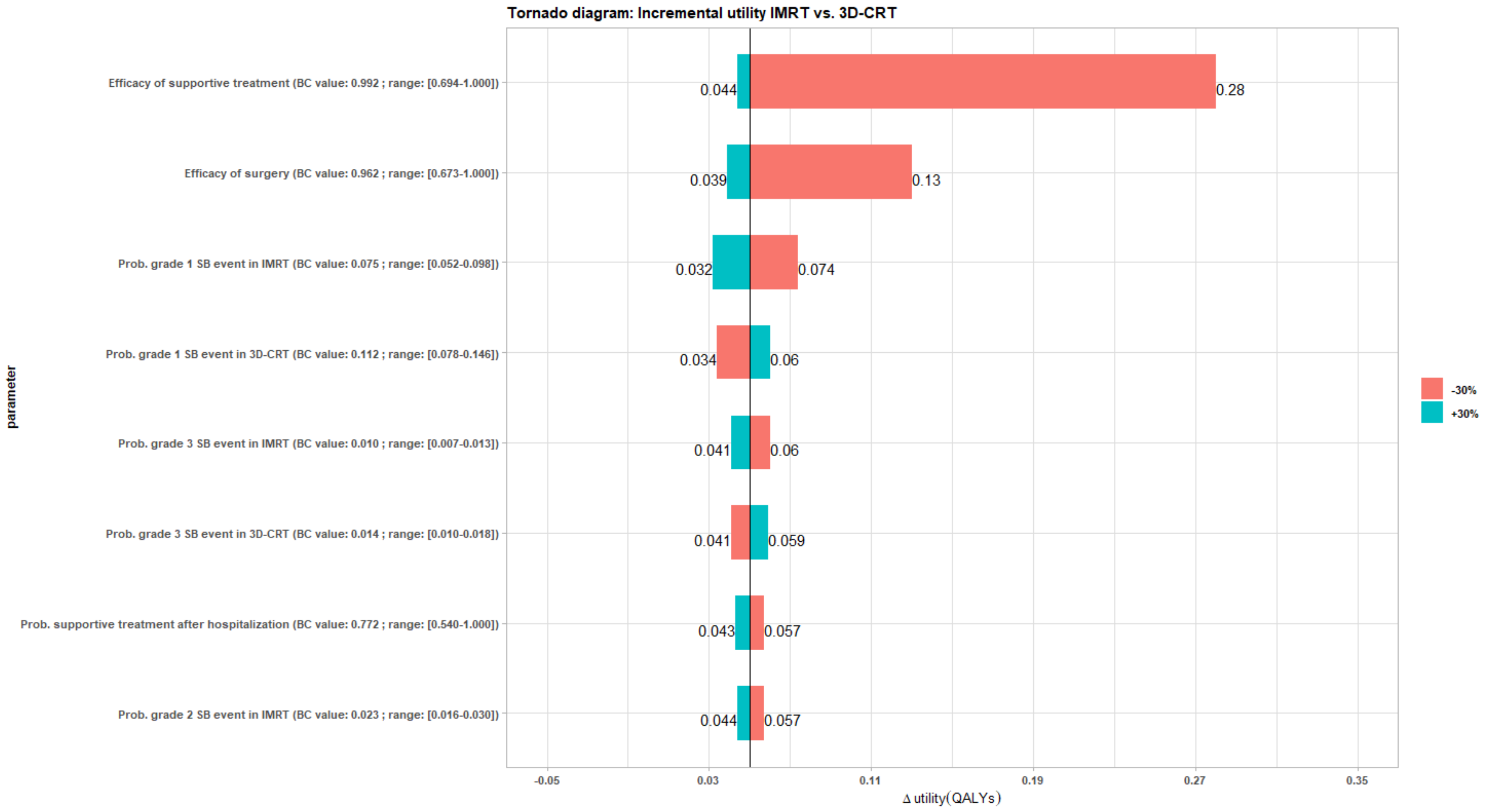
Tornado diagram: Incremental utility IMRT vs. 3D-CRT
parameter
Efficacy of supportive treatment (BC value: 0.992 ; range: [0.694-1.000])
Efficacy of surgery (BC value: 0.962 ; range: [0.673-1.000])
Prob. grade 1 SB event in IMRT (BC value: 0.075 ; range: [0.052-0.098])
Prob. grade 1 SB event in 3D-CRT (BC value: 0.112 ; range: [0.078-0.146])
Prob. grade 3 SB event in IMRT (BC value: 0.010 ; range: [0.007-0.013])
Prob. grade 3 SB event in 3D-CRT (BC value: 0.014 ; range: [0.010-0.018])
Prob. supportive treatment after hospitalization (BC value: 0.772 ; range: [0.540-1.000])
Prob. grade 2 SB event in IMRT (BC value: 0.023 ; range: [0.016-0.030])
0.044
0.28
0.039
0.13
0.032
0.074
0.034
0.06
0.041
0.06
0.041
0.059
0.043
0.057
0.044
0.057
-0.05
0.03
0.11
0.19
0.27
0.35
Δ utility(QALYs)
-30%
+30%
